# Biochemical and biological studies of irradiated and non-irradiated extracts of *Solanum aculeastrum* Dunal fruit

**DOI:** 10.1038/s41598-024-73531-4

**Published:** 2024-10-22

**Authors:** Asmaa A. Amer, Ahmed A. F. Soliman, Walaa A. Alshareef, Yasmine M. Mandour, Mohamad T. Abdelrahman

**Affiliations:** 1 Department of Pharmacognosy, Pharmaceutical and Drug Industries Research Institute, National Research Centre, Dokki, 12622 Cairo, Egypt; 2 Microbiology and Immunology Department, Faculty of Pharmacy, October 6 University, Giza, Egypt; 3 School of Life and Medical Sciences, University of Hertfordshire Hosted by Global Academic Foundation, New Administrative Capital, 11578 Cairo, Egypt; 4 Radioisotopes Department, Nuclear Research Centre, Egyptian Atomic Energy Authority, Cairo, Egypt

**Keywords:** Cancer, Drug discovery, Microbiology, Molecular biology, Plant sciences, Medical research

## Abstract

This study explores the impact of γ-irradiation on ethanolic extracts of *Solanum aculeastrum* Dunal. The anti-cancer and antimicrobial properties were investigated. The obtained results revealed that total phenol (TP) and total flavonoid (TF) of total ethanol extract (100%) (FTE) were higher than 70% ethanol extract (SE), and these contents increased after gamma radiation with 5 kGy. The results of minimum inhibitory concentration (MIC) and minimum bactericidal concentration (MBC) of the *Solanum aculeastrum* extracts suggested that FTE and 5 kGy-irradiated FTE can be used to control and prevent skin infections caused by MRSA and endocarditis, urinary tract infections, and prostatitis caused by *Enterococcus faecalis*. The FTE sample irradiated at 5 kGy showed cytotoxicity for A431 and Hct-116 cell lines similar to the control sample and higher than the toxicity revealed by the samples irradiated at 10 kGy. In normal cells (Bj-1), the toxicity was decreased after irradiation (IC50 = 31 μg/ml) compared to the non-irradiated extract (IC_50_ = 26.1 μg/ml). Molecular docking suggested Sortase A to play a role in chlorogenic acid antibacterial activity towards *Staphylococcus aureus*. In conclusion, γ-irradiation can be used to enhance the phytoconstituents of *Solanum aculeastrum* fruit extracts and, consequently, its biological properties.

**Supplementary Information:**

The online version contains supplementary material available at 10.1038/s41598-024-73531-4.

## Introduction

Medicinal plants provide alternative remedies with medicinal properties capable of mitigating, treating, or preventing a wide range of human diseases, particularly in rural regions^1,2^. Gamma radiation is a widely utilized technique for decontaminating medicinal plants owing to its high effectiveness^3^. The Codex Alimentarius Commission approved this method in 1983 to diminish microbial contamination on plants; therefore, it has been extensively employed in decontaminating herbs and spices^4,5^. The impact of gamma radiation on the cellular structure and active constituents of herbal drugs varies depending on different factors such as dosage, radiosensitivity, plant types, and the physiological state of the plants^6^.

Certain antibiotics may not be effective against some bacteria owing to their natural resistance, while others develop resistance through genetic mutation. The misuse and overuse of antibiotics accelerated the widespread development of antibiotic-resistant bacteria, which led to ineffective treatment because of many resistance traits acquired by bacteria^7^. The most common antibiotic-resistant pathogens are Methicillin-Resistant *Staphylococcus aureus* (MRSA), Vancomycin-Resistant *Enterococcus* (VRE), MultiDrug Resistant *Mycobacterium tuberculosis* (MDR-TB), and Carbapenemase-Producing Enterobacterales (CPE)^8^. The World Health Organization (WHO) 2017 published that *Enterococcus faecium* and *Staphylococcus aureus* represent the high-priority category, exhibiting resistance to multiple antibiotics, including vancomycin and fluoroquinolones^9^.

*Solanum aculeastrum* Dunal, family Solanaceae, is a small tree native to South Africa^10^. It has several synonyms, such as soda apple, soda apple nightshade, goat apple, and goat bitter apple. The fruit of *S. aculeastrum* looks lemon-shaped, having a yellow-green color after ripening^11^. Traditionally, in rural South Africa, leaves and fruit decoction are taken orally to treat stomach disorders and indigestion and as an anti-cancer (12) and anti-Leishmania^12^. Meanwhile, berries (fresh and boiled) are used in the treatment of gonorrhea^13^, jigger wounds, and acne^14,15^. *S. aculeastrum* fruit methanol extract was reported to have an inhibiting activity against some gram-positive and gram-negative bacteria. *Aspergillus niger*,* Candida albicans*,* and Fusarium oxysporum* were the most resistant organisms^16^. Genus Solanum was reported to be rich in steroidal glycoalkaloids and sesquiterpenoids such as solamargine, *β*-solamarine, and solaculine that have antibacterial and antimycotic properties^17,18^. Two steroid glycosides, tomatidine, and solasodine, were isolated from the methanol extract of *S. aculeastrum* and identified by NMR spectroscopic technique^17,19^. They were reported to have activity on HeLa, MCF7, and HT29 cancer cell lines^17,20^.

Till now, there is no report on the type and structure of the phenolic phytoconstituents from the fruit of this plant. This study aimed to investigate the phenolic constituents of the *S. aculeastrum* fruit ethanol extracts and estimate their antioxidant, antimicrobial, and cytotoxic activities before and after exposure to gamma radiation.

## Results

### Phytochemical analysis

#### Phytochemical screening

The preliminary phytochemical screening tests of *S. aculeastrum* (Table 1) showed the existence of different categories of phytoconstituents such as carbohydrates, steroids, phenolics, flavonoids, alkaloids, saponins, proteins/amino acids, and traces of tannins in the FTE and SE extracts.

**Table 1:** Pilot phytochemical screening of the total ethanol extract (70%) of *Solanum aculeastrum* extract.

Phytochemical constituents	Results	
	FTE	SE 70%
Reducing sugars (carbohydrates)	++	++
Steroids and/ or Terpenoids	+++	+++
Flavonoids	++	++
Phenolics	+++	+++
Tannins	±	±
Alkaloids	++	++
Cardiac glycosids	−	−
Proteins/Amino acids	++	++
Anthraquinones	−	−
Saponins	±	±

+++ Abundant.

++ Moderate amounts.

± Traces.

− Absent.

#### Total phenolic, Total flavonoid, and total carbohydrate contents of *Solanum aculeastrum* fruit extracts

The total phenolic, flavonoids, and carbohydrate contents (Table 2) were estimated using gallic acid, rutin, and glucose as standards, respectively. The linear regression equation was used to calculate them from the standard calibration curve.

**Table 2:** Total phenol and total flavonoid contents of *Solanum aculeastrum* extracts.

Fraction	Total phenolic (µg GAE/mg extract)	Total flavonoids (µg RE/mg extract)	Total carbohydrates (µg glucose /mg extract)
FTE	240.53	25.16	264.268
SE	214.19	19.05	187.927
5 KGy FTE	252.17	26.92	
10 KGy FTE	200.89	14.70	

The total phenolic content of FTE, SE, and the irradiated FTE extract with 5 kGy and 10 kGy were 240.53, 214.19, 252.17, and 200.89 µg GAE/mg extract, respectively. While total flavonoids for the same extracts were 25.16, 19.05, 26.92, and 14.70 µg RE/mg extract. The total carbohydrate contents of FTE and SE were 264.268 and 187.927 µg glucose/mg extract, respectively.

### HPLC analysis of total ethanol extract

From the HPLC analysis of the total ethanol extract (FTE) of *S. aculeastrum* fruit (Table 3), thirteen compounds were detected, including eight phenolic acids and two flavonoids. Chlorogenic acid is the major one (62.1929%), followed by gallic acid (11.9483%).

After the gamma irradiation with 5 kGy, ten compounds were identified with a high increase in the concentration of chlorogenic acid (86.7118%), a moderate increase in the concentration of methyl gallate (4.5942%), and a minor increase in other concentrations. The gallic acid concentration was decreased (6.5575%). Quercetin appeared after irradiation; it means that some compounds can be formed while syringic acid disappeared.

After the exposure of the FTE to gamma irradiation with 10 kGy, eleven compounds were detected. Chlorogenic acid (87.9415%) was increased in the concentration. Caffeic and cinnamic acids appeared. Quercetin also appeared but at a lower concentration than after 5 kGy exposure. The gallic acid concentration was also decreased (6.3693%) but was particularly equal to 5kGy. It is considered the first time the investigation of the phenolic constituents of*S. aculeastrum* fruit was conducted.

### Identification of the isolated compounds from FTE extract

**Fig. 1:**
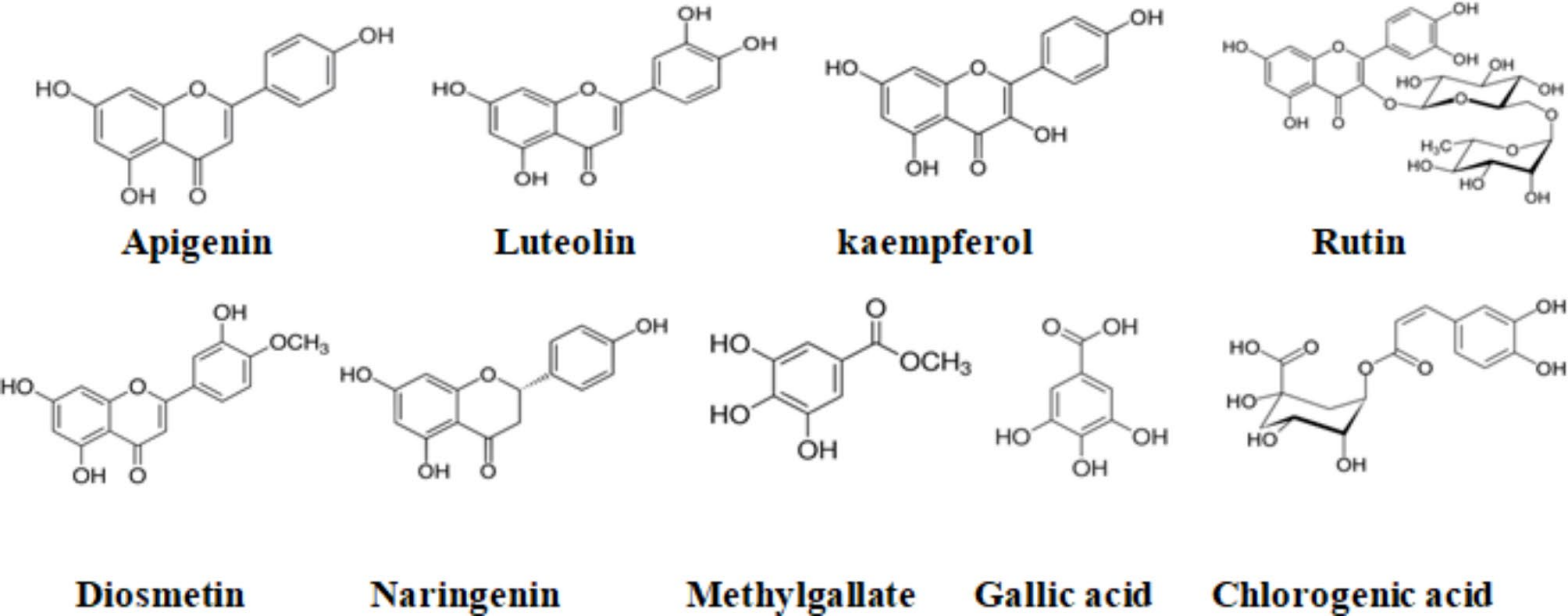
Isolated compounds From *Solanum aculeastrum* Fruit extract (FTE).

Chromatographic and spectroscopic investigation resulted in the isolation and identification of nine compounds (Fig. 1). Gallic acid (1), chlorogenic acid (2), methyl gallate (3), diosmetin (4), naringenin (5), apigenin (6), luteolin (7), kaempferol (8), and rutin (9) were isolated. The identification of compounds (1–5) was done according to physical and chemical properties, UV spectrophotometric analysis, mass spectrometry (EI/MS), and chromatographic data ^1^H and ^13^C-NMR and Co-PC with standard samples). In contrast, compounds (6–9) were identified by UV spectrophotometry and Co-PC with standard samples. It is the first time to isolate these compounds from this species, *S. aculeastrum*. All isolated compounds were detected by HPLC analysis, which approved the presence of these compounds.

**Chlorogenic acid**: white powder, soluble in methanol, m.p. 208–209 °C. It gave yellowish-green fluorescence after the exposure of ammonia solution, which turned to blue fluorescence after spraying with a ferric chloride reagent. 1H-NMR (DMSO-*d*_*6*_, 400 MHz) (δ ppm): δ 6.98 (1H ,*d*, *J* = 8.1, H-5′), 6.78 (1H, *d*, *J* = 8.1, H-6′), 7.04 (1H, br-s, H-2′), δ 7.44 (1H, *d*, *J* = 15.9 Hz, H-7′), 6.17 (1 H, *d*, *J* = 15.9 Hz H-8′), 5.08 (1H, *m*, H-3), 3.57 (1H, *d*, *J* = 7.2, H-4), 3.93 (1H, *br-s*, H-5) and 1.95–2.03 (4H, *m*, H-2 and H-6). The EI-Ms spectrum showed the molecular ion peak (M^+^) at m/z 354.34. The UV spectrum showed that λ max nm (MeOH) (218, 245, 299 (sh.), 329) and (220, 262, 311 (sh.), 373) with NaOMe indicate band I that is characteristic and confirmed a phenolic acid skeleton. It is also called a cinnamic acid derivative (5-(3,4-dihydroxycinnamoylquinic acid).

**Methyl gallate**: off-white amorphous powder, m.p. 201–204 °C, soluble in methanol. The UV spectral data gave peaks at 220 and 288 in methanol and 220 and 325 after adding sodium methoxide (NaOMe). The ^1^H-NMR spectrum (Acetone-*d*_*6*_, 400 MHz), δ_ppm;_ δ 7 (2 H, *br-s*, H-2, H-6), 3.8 (3 H, *s*, methyl ester). The ^13^C-NMR spectrum (Acetone-*d*_*6*_, 100 MHz), δ ppm 167.28 (C=O), 145.43 (C-3,5), 138.47 (C-4), 109.80 (C-1), 120.30 (C-2,6), 51.46 (CH_3_). EI- Mass spectral data (m/z); 184 (M^+^,72%), 153 (M^+^-OCH_3_, 100%), 125 (M^+^-COOCH_3_, 20%), 79 (10.9%). It is the first time to be isolated from the genus of Solanum. The confirmation was achieved by co-chromatography with a reference sample. Methyl gallate was reported to have different biological activities,

**Gallic acid**: Buff amorphous powder, methanol-soluble, *m.p.* 254–255 °C, The UV spectral data gave a peak (λ_max_) at 272 in methanol. EI-Mass spectral data (m/z); [M-H [^−^ 169.01 m/z. The 1H-NMR spectrum (Acetone-*d*_*6*_, 400 MHz), δ_ppm;_δ 7 (2 H, *s*, H-2, H-6). Confirmation was achieved based on co-chromatography with reference sample and HPLC, as it was the major isolated compound (3,4,5 trihydroxybenzoic acid).

**Diosmetin (4’ methyl luteolin)**: Dark Yellow powder, soluble in methylene chloride: methanol. The UV spectral data gave a peak (λ_max_) at 348 nm and 252,270 (sh.). ^1^H-NMR (DMSO-*d*_6_,400 MHz, ); δ 12.94 (s, 1 H),10.84 (s, 1 H), 9.44 (s, 1 H), 7.53 (d, *J* = 7.6 Hz, 1 H), 7.44 (s, 1 H), 7.09 (d, *J* = 8.4 Hz, 1 H), 6.76 (s, 1 H), 6.44 (s, 1 H), 6.19 (s, 1 H) and 3.86 (3 H, *s*).

**Naringenin**: Faint yellow powder, soluble in methanol, *m.p.* 250–252 °C, the UV spectrum data in methanol exhibited two absorption bands (λ_max_) at 289, 329(sh.) nm and 226 nm. The bathochromic shift in the band, I (∆36) with NaOMe, indicated a flavanone derivative. The ^1^H-NMR (DMSO-*d*_6_, 400 MHz) showed that protons at position 3 resonated as two signals at δ 2.68 (1 H, *dd*, *J* = 17.2, 2.9 Hz, H-3β (eq)) and 3.27 (1 H, *dd*, *J* = 17.2, 12.8 Hz, H-3α(ax)). The peak at δ 5.89 (2 H, *br-s*, overlap, H6/8) show the pattern indicating substituted hydroxy groups at C-5, C-7 of ring (A) The peaks at δ 5.42 (*dd*, *J* = 12.76, 2.52 Hz) representing H-2. The doublet peaks appeared at δ 7.33 (2 H, *d*, *J* = 8.4 Hz, H-2’, 6’) while the other peaks appeared at δ 6.81 (2 H, *d*, *J* = 8.4 Hz, H-3’, 5’) assignable to aromatic protons of ring (B) The ^13^C-NMR spectrum (DMSO-d_*6*_) exhibited a signal at 196.8 (carbonyl (C=O) C-4), six quaternary carbons at 163.9, 167.1, 163.4, 102.2, 129.3, and 158.2 (C-5, C-7, C-9, C-10, C-1’, C- 4’). The six olefinic carbons were at 115.6, 128.8, 96.2& 95.4 (C-3’ and 5’, C-2’ and 6’, C-6, and C-8) and one carbon at 78.9 (C-2).

All isolated compounds, chlorogenic acid, gallic acid, methyl gallate, diosmetin, and naringenin were reported to have different biological activities, such as antioxidant, anti-inflammatory, antitumor, and antimicrobial^21–24^.

**Table 3:** HPLC analysis of *Solanum aculeastrum* fruit extracts.

No.	R_t_	Compounds	Area %		
			FTE	5 kGy FTE	10 kGy FTE
1	2.525	Unknown	8.0371	ND	ND
2	**2.855**	Unknown	**5.3187**	**ND**	**ND**
3	**3.048**	Gallic acid	11.9483	6.5575	6.3696
4	3.762	Chlorogenic acid	**62.1929**	**86.7118**	**87.9415**
5	4.548	Unknown	7.3642	ND	ND
6	4.963	Methyl gallate	3.7832	4.5942*	3.2885
7	5.332	Caffeic acid	ND	ND	0.3374**
8	5.800	Syringic acid	0.0255	ND*	ND**
9	5.931	Pyro catechol	0.5252	0.6145	0.6247
10	7.065	Rutin	0.1351	0.5637	0.5263
11	7.609	Ellagic acid	0.0360	0.1861	0.2110
12	8.328	Coumaric acid	0.1609	0.1731	ND**
13	9.317	Ferulic acid	0.1144	0.1145	0.1622
14	10.142	Naringenin	0.3585	0.1308	0.2823
15	12.678	Querectin	ND	0.1599*	0.1356**
16	14.625	Cinnamic acid	ND	ND	0.1206**
Total			13	10	11

Significant values are given in bold.

### Antioxidant activity

**Fig. 2:**
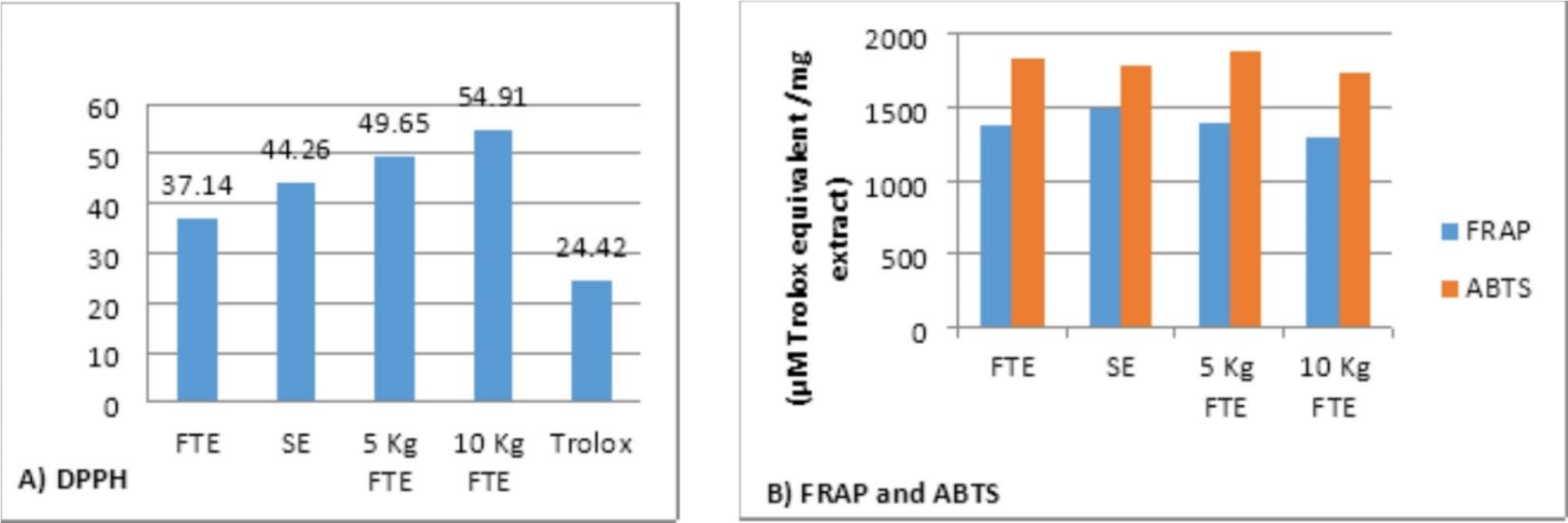
Histogram of the antioxidant activity of the*Solanum aculeastrum*fruit extracts using (**A**) DPPH, (**B**) FRAP and ABTS assays.

The antioxidant capacities of FTE and SE, as well as radiated 5 kGy and 10 kGy, were estimated by means of 3 different complementary methods: DPPH, ABTS and FRAP (Table 4 and Fig. 2). DPPH assay showed that FTE in the highest antioxidant power (IC_50_ 37.14 ± 1.46 μg/ mL), followed by SE, 5 KGy FTE, and, 10 KGy FTE (IC_50_ 44.26 ± 1.23, 49.65 ± 1.42, and 54.91 ± 1.83 μg/ mL), respectively. While FRAP and ABTS assays showed that 5 KGy FTE has the highest antioxidant effect (1396.71 and 1883.39 µM Trolox equivalent /mg extract, respectively), followed by FTE (1383.47 and 1841.32 µM Trolox equivalent /mg extract, respectively). This is the earliest report of the antioxidant activity for*S. aculeastrum* fruit extract by FRAP and ABTS methods.

**Table 4:** Antioxidant activity of the *Solanum aculeastrum* extracts using DPPH, FRAP and ABTS methods.

Antioxidant activity	DPPH IC_50_ (μg/ mL)	FRAP	ABTS
		(µM Trolox equivalent /mg extract)	
FTE	37.14 ± 1.46	1383.47	1841.32
SE	44.26 ± 1.23	1498	1777.53
5 KGy FTE	49.65 ± 1.42	1396.71	1883.39
10 KGy FTE	54.91 ± 1.83	1298	1732.74
Trolox	24.42 ± 0.87	–	–

### Antimicrobial

#### Antibacterial activity of*S. aculeastrum* extracts

**Fig. 3:**
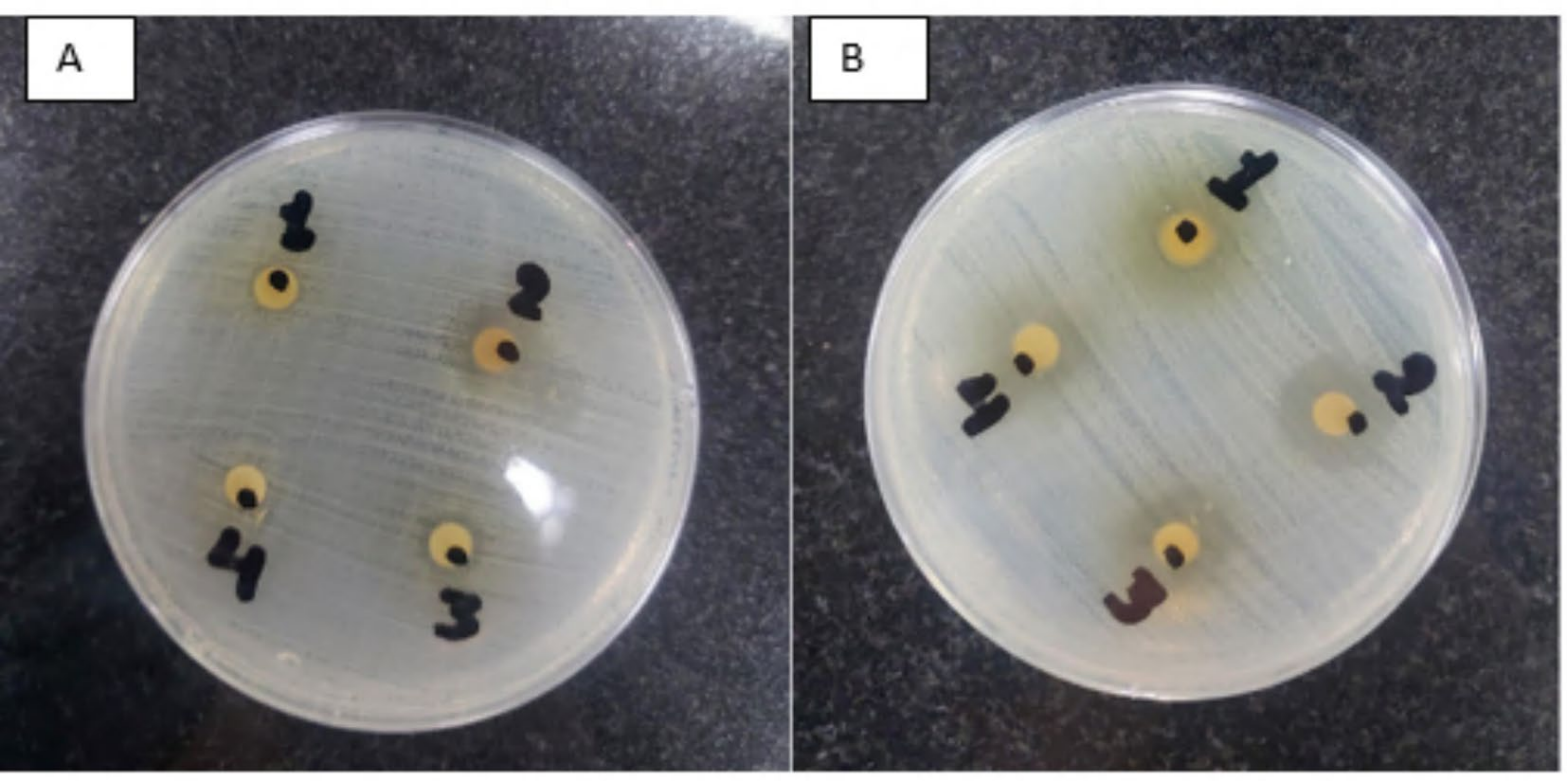
Growth inhibition of tested pathogenic bacterial strains caused by *S. aculeastrum* extracts. (**A**) MRSA growth with 1 (SE; 70%), 2 (FTE; 100%), 3 (5 kGy-irradiated FTE), and 4 (10 kGy-irradiated FTE). (**B**) *E. faecalis* growth with 1 (SE; 70%), 2 (FTE; 100%), 3 (5 kGy- irradiated FTE), and 4 (10 kGy-irradiated FTE).

*S. aculeastrum* fruit extracts were evaluated for its antibacterial activity against two strains of pathogenic G + ve bacteria (MRSA and *E. faecalis*), using the disc diffusion method. The obtained results were recorded and illustrated in Table 5; Fig. 3. The results showed the efficacy of the plant extracts in inhibiting the growth of selected bacteria at variable potency levels. The FTE and 5 kGy-irradiated FTE were the most effective extracts retarding microbial growth of all tested pathogenic bacteria at a concentration 100 mg/ml, while SE and 10 kGy extracts were effective only against *E. faecalis*.

**Table 5:** Antibacterial screening test of* S. aculeastrum* extracts (100 mg/ml) followed by MIC and MBC against MRSA and* E. faecalis* strains.

Strain	ZOI (mm)				MIC (mg/ml)				MBC (mg/ml)			
	70%	100%	5 KGy	10 KGy	70%	100%	5 KGy	10 KGy	70%	100%	5 KGy	10 KGy
MRSA	No ZOI	11	10	No ZOI	ND	50	37.5	*ND	ND	ND	> 50	ND
*E. faecalis*	14	13	11	10	12.5	12.5	25	50	50	> 50	50	ND

*ND (not determined).

Where (SE = 70%), FTE = 100%), 5 KGy irradiated FTE, and 10KGy irradiated FTE.

**Fig. 4:**
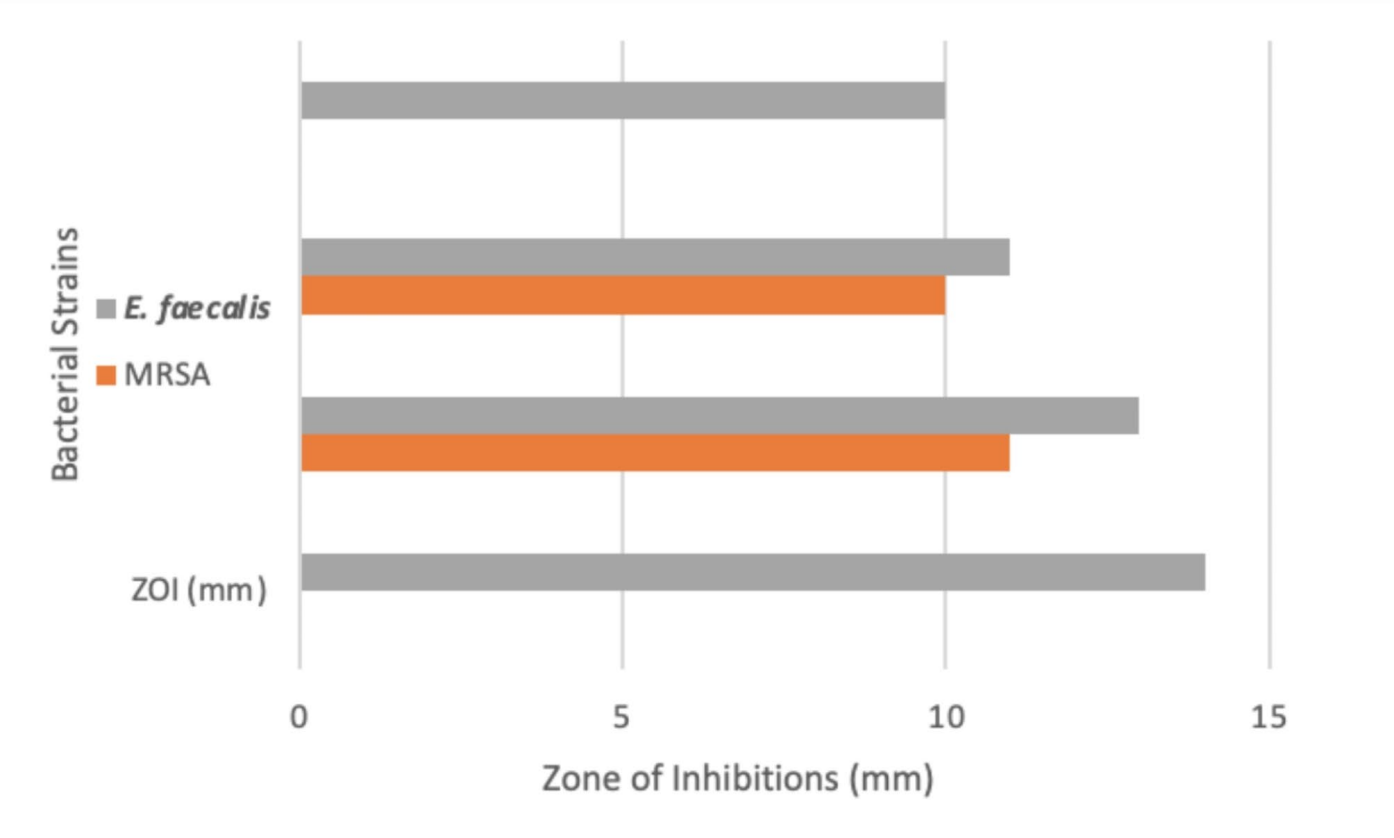
Antibacterial susceptibility of the *S. aculeastrum* extracts against MRSA and *E. faecalis*.

The outcomes of the antibacterial action of all tested extracts suggest that MRSA was a tolerant strain to plant extracts of SE and 10 k Gy irradiated FTE, while *E. faecalis* was the most susceptible strain to the extracted plant contents, as shown in Fig. 4. Therefore, experiments were organized to estimate their minimal inhibitory concentration (MIC) and minimal bactericidal concentration (MBC) on the MRSA and *E. faecalis.*

#### Minimum inhibitory concentrations (MIC) of the effective*S. aculeastrum*extracts

Minimal inhibitory concentration (MIC) values of different extracts from the ***S. aculeastrum*** against the tested bacteria are mentioned in Table 5. The FTE and 5 kGy irradiated FTE extracts inhibited MRSA and *E. faecalis* growth at MIC, ranging between 12.5 and 50 mg/ml. Whereas SE and 10 kGy-irradiated FTE of the extracts did not show any activity against MRSA in contrast to *E. faecalis* which showed MIC 12.5 mg/ml and 50 mg/ml, correspondingly.

#### Minimum bactericidal concentrations (MBC) of the*S. aculeastrum*extracts

The MBC was established by the nonappearance of bacterial growth of the tested strains streaked from the inhibition zone matching their lowermost MICs. SE and 5 kg radiated FTE extracts showed potentially bactericidal activity against the tested pathogenic bacteria (***E.****faecalis*) with MBC of 50 mg/ml while MBC of 100% and 5 kGy reached to > 50 mg/ml against *E. faecalis* and MRSA, respectively.

The results of MIC and MBC of the *S. aculeastrum* extracts suggested that FTE and 5 kGy radiated FTE can be used to control and prevent skin infections caused by MRSA and endocarditis, urinary tract infections, and prostatitis caused by *E. faecalis*. The bacterial strains in this study were chosen based on their engagement and importance in different diseases (mentioned above).

FTE and 5 kGy- irradiated FTE extracts suppress bacterial growth of all tested bacterial strains, followed by an extract of 70%, which appears to be potentially effective against *E. faecalis* strains and less effective against MRSA. It seems to be the first report on these bacterial strains.

### Cytotoxicity

The preliminary screening of two extracts, namely FTE and SE ethanol extracts, examined for cytotoxicity against four human cancer cell lines: A341, MCF-7and HCT-116, and one normal cell line BJ-1. The results showed the FTE extract was the most active extract with the lowest IC50 values of 25.8, 32.8, 31.5, and 23.7 µg/mL against A341, MDA-MB231, MCF-7and HCT-116, respectively. The most active extract was irradiated at 10 KGy and 5 KGy to ensure that preservation of extracts using radiation did not significantly reduce their efficacy (Table 6).

**Table 6:** Cytotoxicity of samples (100 µg/ml) on three human tumor cell lines: skin cancer (A431), breast carcinoma (MCF-)7 and colon carcinoma (HCT-116). And one normal cell line (BJ-1).

	IC50 μg/ml			
	A431	MCF-7	HCT-116	Bj-1
FTE	25.8	31.5	23.7	26.1
SE	25.7	33.3	28.0	42.5
Doxorubicin	13.4	37.6	26.1	28.2

The 5 kGy-irradiated FTE showed cytotoxic activity for A431 and Hct-116 cell lines alike the control, and higher than the toxicity shown by the 10 kGy-irradiated samples. In the case of normal cells (Bj-1), the toxicity was decreased after irradiation (IC_50_ = 31 μg/ml) than the non-irradiated extract sample (IC_50_ = 26.1) (Tables 7, 8 and 9).

**Table 7:** Cytotoxicity of the most active sample (FTE) after irradiation at 5 and 10 KGy.

	IC50 μg/ml			
	A431	MCF-7	HCT-116	Bj-1
FTE 5KGy	15.1	26.9	15.7	31.1
FTE 10KGy	17.1	28.6	18.1	41.9

**Table 8:** In vitro-cytotoxic activity (IC _50_ µg/ml) of samples tested against different cancer cell lines after 48 h.

	Cytotoxicity against four cancer cell line and one normal cell line at 100 μg/ml			
	A431	MCF-7	HCT-116	BJ-1
FTE 5KGy	95.9	92.8	100	94
FTE 10KGy	96	93.3	100	93.6

**Table 9:** In vitro cytotoxic activity (IC _50_ µg/ml) of the most active sample tested; FTE against different cancer cell lines after 48 h.

	Cytotoxicity against four cancer cell line and one normal cell line at 100μg/ml			
	A341	MCF-7	HCT-116	BJ-1
FTE	98.7	96.8	96	97.9
SE	99.5	97	98	99.9
Doxorubicin	99.6	98.9	99.7	89.6

### Molecular modeling

**Fig. 5:**
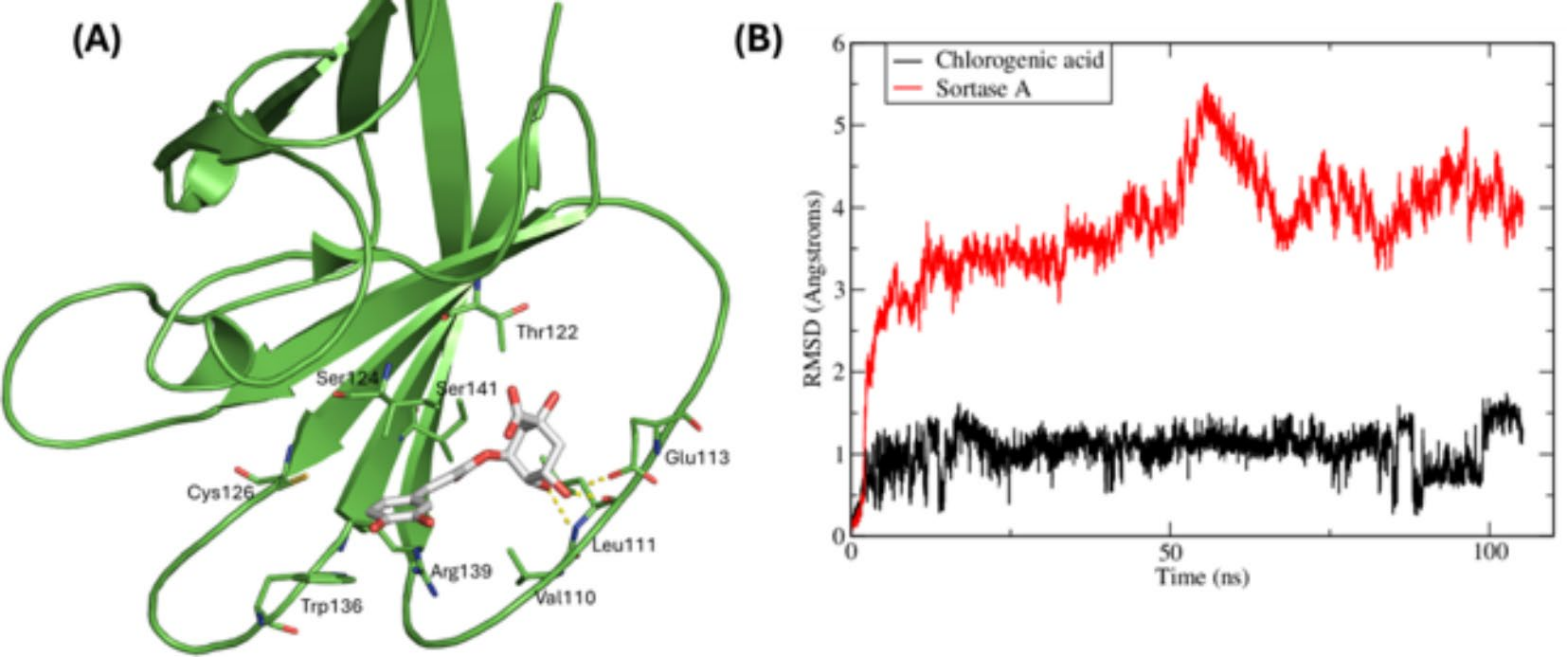
(**A**) Binding mode of Chlorogenic acid (grey, sticks) in the active site of Sortase A (PDB:2MLM) (green, cartoon). (**B**) Root-mean-square deviations (RMSD) of Sortase A backbone atoms (red) and Chlorogenic acid heavy atoms (black) between the trajectory frames and the starting geometry.

*Staphylococcus aureus* is a dangerous pathogen universally linked with high morbidity and mortality numbers. Sortase A (SrtA) has emerged as a hopeful molecular target for developing anti-staphylococcal agents^25^. Chlorogenic acid is the most abundant component in the extract (as revealed by HPLC analysis results in Table 3). A docking experiment of chlorogenic acid (CLA) against SrtA revealed its ability to fill the active site with the phenyl ring of the caffeic acid moiety forming cation-π interactions with the side chain of the basic Arg139 and two of the hydroxyl groups of the L-quinic acid form H-bonds with the backbone atoms of Val110 and Leu111 and the side chain of Glu113 (Fig. 5A). Even though the co-crystallized ligand is a covalent inhibitor, it also formed interaction with Arg139 similar to CLA. A 100-ns MD simulation of SrtA and CLA complex was conducted which showed CLA to remain stable inside the binding site of SrtA. The RMSD fluctuations of SrtA backbone atoms showed the protein to adapt a relatively stable conformation starting 30 ns till the end of the simulation with a slightly large fluctuations of around 1 Å due to loops flexibility (Fig. 5B). On the other hand, CLA showed a very stable binding mode with a plateau observed at 1 Å starting 25 ns.

## Discussion

*Solanum* is the largest genus within the family Solanaceae, comprising 2000 genera. Solanum plants are characterized by their rich steroidal, glycoalkaloids, and sesquiterpenoids^26^. The results obtained from the current study regarding the preliminary phytochemical screening of FTE and SE extracts revealed the presence of phenolics, flavonoids, carbohydrates, steroids, alkaloids, saponins, proteins/amino acids, and traces of tannins. These results are in accordance with the previously published information by Kaunda and Zhang^27^. They showed the presence of phenolic compounds: flavonoids and coumarins, steroidal saponins, steroidal alkaloids, terpenes, and sterol compounds on different *Solanum* species^27^. FTE’s total phenolic and flavonoid (TP and TF) contents were higher than SE, with 1.12 and 1.32 folds, respectively. Both extracts were subjected to cytotoxic examination against four human cancer cell lines, A341, MCF-7, and HCT-116, and one normal cell line, BJ-1, to choose the most active one. The results showed the FTE extract was the most active extract with the lowest IC_50_ values. These results were compatible with the previous results regarding the cytotoxic effect of the leaves and fruit methanol extracts of *S. aculeastrum*^16,27^.

The highest phenolic content and most active extract, FTE, was exposed to gamma irradiation in different doses (5, 10 kGy) as it could affect the yield, chemical constituents, and, therefore, the biological activities of the plant extracts^28^. The phenolic concentrations might be influenced by irradiation with different loads. There wasn’t any previous record of TP, TF, and TC contents of*S. aculeastrum*, as well as the impact of gamma radiation on their contents and biological effectiveness, so it seems that our study is the first record of those amounts and effects.

The TP and TF contents increased after exposure to gamma irradiation with 5 kGy and decreased with 10 kGy. The irradiation of FTE extract increased some compound’s concentration, the appearance of other compounds, and the vanishment of some compounds, which HPLC detected.

HPLC analysis of FTE detected thirteen compounds, including eight phenolic acids and two flavonoids. Chlorogenic acid is the major one, followed by gallic acid. After the exposure to 5 kGy and 10 kGy gamma irradiation, more compounds were identified in addition to the previous compounds. Notably, there was a high increase in the chlorogenic acid concentration and a decrease in the gallic acid concentration with both doses. Quercetin was detected in both doses but in large quantities with 5 kGy. At the same time, caffeic and cinnamic acids were detected with 10 kGy only. In contrast, there was a moderate increase in the methyl gallate concentration and a minor increase in other concentrations with the disappearance of syringic acid after 5 kGy.

The chromatographic separation and spectroscopic identification of phenolic compounds resulted in nine compounds: gallic acid, chlorogenic acid, methyl gallate, diosmetin, naringenin, apigenin, luteolin, kaempferol, and rutin. It is the first report on the phenolic constituents of the total ethanol extract of this species, *S. aculeastrum*. Apigenin, luteolin, kaempferol, and rutin were previously identified in other species of *Solanum*^27^. Gallic acid, chlorogenic acid, and rutin were previously isolated from the fruit of another species, *Solanum anguvi*^29^. Phenolics were reported to be responsible for many biological activities: anti-oxidant, antimicrobial, anti-inflammatory, and anticancer^30–32^. Chlorogenic acid (CLA), the major compound in FTE, was reported to have highly inhibitory effects^33^, antitumor, and liver and kidney protector drug^22^.

All extracts, FTE and SE, and radiated FTE with 5 kGy and 10 kGy were subjected to biological evaluation against the antioxidant, antimicrobial, and cytotoxic activity. According to the antioxidant examination, FTE showed a higher antioxidant power with the DPPH assay, while 5 KGy FTE showed the highest redox effect with FRAP and ABTS assays.

Regarding the cytotoxic activity, the 5 kGy-irradiated FTE showed cytotoxic activity for A431 and Hct-116 cell lines better than 10 kGy-irradiated samples, also, the toxicity on the normal cells (Bj-1) was decreased after irradiation than the non-irradiated extract sample. The biological activities of the fruit extracts may be due to the presence of different phytoconstituents not only phenolic constituents. It was reported that the steroid glycosides, tomatidine, and solasodine separated from the CH_2_Cl_2_/MeOH extracts had significant anticancer effects against HeLa, MCF7, and HT29 cell lines^20,34^.

It was reported that *S. aculeastrum* methanol and water extracts of the fruits were investigated against Gram-positive and Gram-negative bacterial; *Bacillus cereus*,* Staphylococcus epidermidis*,* Staphylococcus aureus*,* Micrococcus kristinae*,* Streptococcus pyogenes*,* Escherichia coli*,* Salmonella pooni*,* Serratia marcescens*,* Pseudomonas aeruginosa*,* Klebsiella pneumonia*. Methanol extracts inhibited the growth of both at different MICs, oscillating from 4.0 to 10.0 mg/ml, which were more active against Gram-positive bacteria. However, water extracts of both did not have any activity against each of them. Neither methanol nor aqueous extracts of the fruits or leaves revealed any action on *Aspergillus niger*,* Fusarium oxysporum*,* and Candida albicans*^16^.

The spread of serious infectious diseases and their effective treatment have become risky in light of antibiotic-resistant microorganisms. WHO released a list in 2017 that categorizes 12 families of antibiotic-resistant bacteria into 3 categories regarding their priority to develop novel antibiotics into critical, high, and medium priority^35^. The critical priority includes *Acinetobacter*, *Pseudomonas*, *E. coli*, and some *Enterobacter* spp. Meanwhile, *Enterococcus faecium* and *Staphylococcus aureus* are represented in the high-priority category^8,9^. Furthermore, there is a need for developing new antibiotics to counteract these harmful microorganisms^36^.

MRSA and *E. faecalis*, gram-positive bacteria, are considered major microorganisms of hospital-acquired infections, coupled with noteworthy mortality, morbidity, and duration of residency. Skin and subcutaneous tissues are the most common infectious organs with MRSA, followed by meningitis, pneumonia, osteomyelitis, lung abscess, and empyema^37,38^. *E. faecalis* causes several infectious human diseases, such as urinary tract infections, bacteremia, wound infections, and endocarditis^38^. In the current study, the growth of MRSA and *E. faecalis* was inhibited by the FTE and 5 kGy irradiated FTE extracts at MIC, ranging between 12.5 and 50 mg/ml. FTE is the major inhibitory agent for MERSA (50 mg/ml), followed by 5 kGy (37.5 mg/ml), while 5 kGy is the major inhibitory against E. faecalis (25 mg/ml), followed by FTE (12.5 mg/ml). This result is in agreement with the published data by Elward et al. and Ash, who mentioned that the MIC of antibiotics against MRSA and *E. faecalis* should be ≥ 4 µg/mL based on the antibiotic susceptibilities tests^39,40^. These activities may be attributed to the phenolic constituents, especially chlorogenic acid.

However, *Staphylococcus aureus* is a dangerous pathogen universally linked with high morbidity and mortality numbers. Sortase A (SrtA) has emerged as a hopeful molecular target in treating MRSA^25^. In the current work of the docking study, the extracts showed a promising ability to inhibit MRSA (*Staphylococcus aureus*), while previous studies pointed out the potential of chlorogenic acid to inhibit MRSA by inhibiting SrtA through in-vivo study^41,42^.

Dihydrofolate reductase (DHFR) is an enzyme converting dihydrofolate into tetrahydrofolate using NADPH^43^, which is vital in synthesizing nucleotides and proteins^44^. Docking of chlorogenic acid showed its capacity to harbor the active site of *E. faecalis* in a similar manner to its ligand NADP. This result agreed with the wet lab result, where the extract (having CLA as a major component) could inhibit *E. faecalis*. This is the first report on the inhibitory effect of chlorogenic acid on *E. faecalis.*

From all obtained data and previously published results, we could conclude that *Solanum aculeastrum* fruits are rich in phenolic compounds isolated for the first time from this species. Phenolic compounds are responsible for antioxidant, antimicrobial, and anticancer activities. The total ethanol extract, FTE, and 5 kGy radiated FTE show promising antimicrobial activity against MRSA and *E. faecalis*, antibiotic-resistant pathogens. Furthermore, FTE is more active as an antioxidant, and has an anti-cancer effect. Gamma rays enhanced these activities, and 5 kGy radiated FTE is more active against skin cancer cell lines. So, extracts derived from *Solanum aculeastrum* Dunal fruit (FTE and 5 kGy FTE) are suggested as an ideal antibiotic against resistant bacteria, MRSA, and *E. faecalis.* In addition to their antioxidant and also as a topical anticancer drug for skin cancer to a limit and continue with another drug not affected on normal cells. This is the first report evaluating the biological effects of these extracts.

## Methods

### Plant material

Fresh ripe berries of wild plant *S. aculeastrum* were collected from Kisumu County (Kenya) in July 2019. Berries were identified at the Chiromo Campus herbarium, University of Nairobi, compared to reference voucher specimen LLT 2012/001. Prof. Dr. Wafaa M. Amer, Cairo University Herbarium, Faculty of Science (CAI), checked and identified the specimens The collection of plant material complied with relevant institutional, national, and international guidelines and legislation.

### Preparation of extract

Fresh fruits (800 g) were washed with tap water, dried with tissues, chopped, and divided into two portions. The first one (400 g) was extracted with 100% ethanol (FTE) three times (3 L) until exhaustion, and the second portion (400 g) was soaked in 70% ethanol (SE) until exhaustion. Buchner funnel and Whatman No. 1 filter paper (Maidstone, UK) were used to filter extracts and then concentrated using a rotary evaporator with vacuum.

### Irradiation

In this experiment, the extracts were exposed to gamma radiation at the Nuclear Research Center in Inshas, Egypt. A Cobalt-60 (60Co) source was used in a gamma irradiation cell at a dosage rate of 476.26 Gy/hr to achieve the doses (5 KGy, 10 KGy).

### Phytochemical analysis

#### Qualitative phytochemical screening

FTE and SE were subjected to phytochemical screening for plant secondary metabolites: terpenoids/steroids, carbohydrates, flavonoids, alkaloids, saponins, tannins, proteins/amino acids, cardiac glycosides, and anthraquinones. The results of these tests were the observation of the color intensity and any precipitate formed^45,46^.

#### Quantitative estimation of Total Phenolic (TP), flavonoids (TF), and soluble carbohydrate (TSC) contents of FTE

Total phenolic and total flavonoid contents were evaluated according to published methods^47–49^. Gerhardt et al.^50^ method was used to determine total soluble carbohydrates. Measurement was performed at ℷ630, 420, and 578 nm for TP, TF, and TSC, respectively. Results for TP and TF were signified as gallic acid (GAE) and rutin (RE) equivalents, respectively, as discussed by Attard^51^. In contrast, TSC was presented as a *µ*g glucose/mg sample.

#### HPLC analysis of FTE, *R5kGy*, *R10KGy*

HPLC analysis was carried out for the most active extract, FTE, and the same extract under different radiation capacities, 5 kGy, and 10 kGy, using an Agilent system (Agilent Technologies 1260 series, Germany) attached to a diode array detector. This analysis estimated the phenolic compounds based on their retention time and UV spectra. The separation process was performed according to the methods discussed^48^ in the supplementary file S1.

### Separation and identification of compounds of FTE

Two grams of FTE were subjected to preparative separations at a PuriFlash 4100 system; Interchim Software 5.0 (Interchim; Montluçon, France) attached to a PDA-UV-Vis detector 190–840 nm. The separation, fractionation, and isolation of compounds^52–54^ were detailed in the supplementary file S1.

### Biological study

The biological study was done under approval no. (321222021) from the Medical Research Ethics Committee (MREC) of the National Research Centre (NRC-Egypt).

### Antioxidant activity

All samples, FTE and 5 and10 kGy FTE, were subjected to three different assays (DPPH, ABTS, and FRAP) to evaluate the antioxidant power^47^. The DPPH (1, 1-diphenyl-2-picrylhydrazyl) assay was carried out according to the method of Boly et al.^55^. It depends on the reduction in the color intensity of DPPH, which was measured at 540 nm^48,56^. Data were represented as IC_50_ values. The higher effectiveness of the antioxidant power was expressed as lower IC_50_ values. Samples were analyzed in triplicate.

The Ferric Reducing Antioxidant Power (FRAP) assay was used to measure the total antioxidant capacity of *S. aculeastrum* utilizing the method of Benzi et al.^57^, with minor modifications. The assay is based on the ferric-reducing ability of the antioxidants present in the samples. While the ABTS (2,2′-azinobis-(3-ethylbenzothiazoline-6-sulfonate) assay was performed according to the method described by Arnao et al.^58^. The results of the FRAP and ABTS•+ radical assays were expressed as *µ*M TE/mg sample (Trolox equivalent antioxidant capacity (TEAC)) using Trolox as a reference standard. For detailed procedures, see the supplementary file S1.

### Antimicrobial activity

#### Antibacterial susceptibility test of *S. aculeastrum*

The ATCC 29212 was obtained from the Microbiology and Immunology Department, October 6 University, Egypt.

*Methicillin-Resistant Staphylococcus aureus* (MRSA) and *Enterococcus faecalis* organisms were maintained on Muller-Hinton Agar (MHA) plates. They were recovered for testing by growth in Muller-Hinton broth for 24 h at 37 °C. Before use, each bacterial culture was adjusted to match the optical density of a 0.5 McFarland standard (1.5 × 10^7–8^ CFU/ml)^59^. In this study, the disc diffusion test, the Kirby-Bauer method, was used to detect the susceptibility of the *S. aculeastrum* extracts against MRSA and *E. faecalis.* The inhibition zone size inversely correlates with MIC^60^.

#### Determination of minimum inhibitory concentrations (MIC) of the effective *S. aculeastrum *extract

The minimal inhibitory concentration (MIC) is the lowest concentration of an antimicrobial agent that suppresses microbiological growth after 24 h of incubation. The most potent plant extracts demonstrating potent antibacterial activity at a concentration of 100 mg/ml were modified to determine their minimal inhibitory concentration (MIC) and evaluate their efficacy in reducing the pathogenicity of bacterial strains. The inhibition zones were measured using a Vernier caliper and recorded in relation to the effective plant extract concentrations^61^. For more details, see the supplementary file S1.

#### Determination of minimum bactericidal concentrations (MBCs) of the effective *S. aculeastrum *extract

From the two lowest doses of plant extract plates with no observable growth (from the inhibition zone of MIC plates), streaks were extracted and subcultured onto sterile Tryptone soy agar (TSA) plates. Plates were incubated at 37 °C for 24 h and then analyzed for bacterial growth in proportion to the concentration of plant extract. The minimal bactericidal concentration (MBC) was determined as the concentration of plant extract that did not support bacterial growth on freshly infected agar plates^62^.

### Cytotoxic activity

#### Cell culture

A431 (cervix squamous cell carcinoma), HCT-116 (colorectal carcinoma), MCF-7 (breast adenocarcinoma), and one normal fibroblast cell (BJ-1) were kept in RPMI and DMAM F12 for normal cells. Feal bovine serum (10%) was supplied to all media and incubated at CO_2_ (5%) and humidity (95%). Cells were sub-cultured using trypsin (0.15%). All cancer cell lines were purchased from Viscera (Giza, Egypt). While, Skin normal human cell line (BJ-1) immortalized normal foreskin fibroblast cell line was kindly provided by Professor Stig Linder, Oncology and Pathology department, Karolinska Institute, Stockholm, Sweden.

#### Cell viability assay

The procedure assay^63^ was illustrated in detail in the supplementary file S1.

#### Determination of IC50 values

Different concentrations of highly active samples possessing ≥ 75% cytotoxicity on various cancer cell lines were prepared for dose-response studies. For more details, see the supplementary file S1.

#### Molecular docking

The X-ray protein structure of sortase A (SrtA) from *the Staphylococcus aureus* complexed with a covalent inhibitor was retrieved from the protein data bank website (http://www.rcsb.org) (PDB ID: 2MLM)^64^. The structure of SrtA was protonated and that of Chlorogenic acid (CLA) was protonated and minimized using MOE^65^. Docking experiments were conducted using GOLD software^66,67^ as previously described^68,69^ using ChemPLP scoring method.

#### Molecular dynamics

A 100-ns MD simulation study was conducted for the SrtA-CLA complex in a simulated water box using the AMBER software package^70^ as previously described^71^. Analysis was conducted using CPPTraj^72^ and figures generated using XMgrace^73^ and Pymol^74^.

## Electronic supplementary material

Below is the link to the electronic supplementary material.

Supplementary Material 1

## Data Availability

All relevant data are within the paper and its supplementary information files. Any other data would be available from the corresponding author upon reasonable request.
