# Supplementary material for: Biochemical and biological studies of irradiated and non-irradiated extracts of Solanum aculeastrum Dunal fruit
Source: Sci Rep. 2024 Oct 22;14:24829. doi: 10.1038/s41598-024-73531-4 (PMC11496676; doi:10.1038/s41598-024-73531-4)
Supplement: Supplementary file 1 — Supplementary Material 1 [file 41598_2024_73531_MOESM1_ESM.pdf]

Methods

Phytochemical analysis

HPLC analysis of FTE, *R5kgy, R10Kgy*

The separation process was carried out using Eclipse C_{18} column (4.6 mm * 250 mm i.d., 5 μm). The mobile phase consisted of water (A) and 0.05% trifluoroacetic acid in acetonitrile (B) at 1 ml/min flow rate. The mobile phase was programmed consecutively in a linear gradient as follows: 0 min (82% A); 0–5 min (80% A); 5-8 min (60% A); 8-12 min (60% A); 12-15 min (82% A); 15-16 min (82% A) and 16-20 (82%A). The injection volume was 5 μl for each of the sample solutions. The multi-wavelength detector was monitored at 280 nm. The column temperature was 40 °C.

Separation and identification of compounds of FTE

The separation was carried out using a C_{18}-HP column (30 μm). The mobile phase consisting of water (A) and acetonitrile (B) was programmed in a gradient elution. The Process led to 50 fractions which were inspected by chromatographic paper chromatography 1MM (CPP IMM) and using acetic acid 15% and Butanol: Acetic acid: water (BAW) 4:1:5 as running system. The similar fractions were combined to obtain twelve substantial fractions (sub-fractions I–XII). These fractions were subjected to different chromatographic techniques including 3MM preparative paper chromatography and Sephadex LH-20 column using eluents of different polarities this led to the isolation and purification of five compounds. The isolated compounds were structurally elucidated through different investigation; physical, chemical, chromatographic and spectral data (UV, NMR, and MS) (54-56).

Biological study

Antioxidant (DPPH, ABTS, FRAP)

Reducing Power (FRAP assay, Ferric reducing Antioxidant Power)

Briefly; 10 µL of the sample were mixed with 190 uL from the freshly prepared 2,4,6-tris(1-pyridyl)-5-triazine (TPTZ) reagent in 96 wells plate (*n*=3). After the incubation for 30 min in dark at room temperature, the blue color formed was measured using microplate reader FluoStar Omega at 593 nm. Data are represented as means ± SD. The increase in iron reduction was expressed as increase in the absorbance and presented as μM TE/ mg sample (Trolox equivalent per milligram sample).

ABTS assay

The ABTS (2,2'-azinobis-(3-ethylbenzothiazoline-6-sulfonate) assay was carried out according to the method described by Arnao *et al*. (60). with minor modifications to be carried out in microplates. ABTS assay measures the relative ability of antioxidants

to scavenge the ABTS generated in an aqueous phase, as compared with a Trolox (water-soluble vitamin E analogue) standard. Briefly; ABTS (192 mg) was dissolved in distilled water and transferred to a volumetric flask (50 mL), then the volume was completed with distilled water. 1mL of the previous solution was added to 17 μL of 140 mM potassium persulphate and the mixture was left in the dark for 24 h. After that, 1 mL of the reaction mixture was completed to 50 mL with methanol to obtain the final ABTS dilution used in the assay. Freshly prepared ABTS reagent (190 μL) was mixed with the sample (10 μL) in 96 wells plate (*n*=6), the reaction was incubated at room temperature for 120 min in dark. At the end of incubation time, the decrease in ABTS color intensity was measured at 734 nm. Data are represented as means ± SD according to the following equation:

$$Percentage\ inhibition = \left(\frac{Average\ absorbance\ of\ blank - average\ absorbance\ of\ the\ test}{Average\ absorbance\ of\ blank}\right) * 100$$

The total antioxidant capacities were expressed as trolox equivalent antioxidant capacity (TEAC). The effective antioxidant activity could be determined by TEAC. There is a direct relation between the antioxidant activity of the samples and TEAC

2.5.2. Antimicrobial activity

Determination of minimum inhibitory concentrations (MIC) of the effective *Solanum aculeastrum* extract

Different concentrations of the effective plant extract (100 mg/ml) were made by dissolving 300 mg of plant material in 3 ml of WFI, followed by sterilizing the solution with a Millipore filter, and loading the required amount onto sterilized filter paper discs (8 mm in diameter). Mueller-Hilton Agar was placed into sterile Petri dishes, and bacterial suspensions of pathogenic strains were planted into the agar. On top of the MHA plates were placed filter paper discs with various amounts of an efficient plant extract. The plates were kept in the fridge at 5 °C for 2 h to permit plant extract diffusion, then incubated at 37 °C for 24 h. The inhibition zones were measured using a Vernier caliper and recorded in relation to the effective plant extract concentrations(63).

Cytotoxic activity

Cell viability assay

After 24 h of seeding 20000 cells per well in case of HCT-116 and Normal cells, 10000 cells per well in case of MCF-7, and A431 cell lines (in 96 well plates), the medium was changed to serum-free medium containing a final concentration of the extracts of 100 µg/ml in triplicates. The cells were treated for 48 h. 100 µg/ml doxorubicin was used as positive control and 0.5 % DMSO was used as negative control. Cell viability was determined using the MTT (3-(4, 5-dimethylthiazol-2-yl)-2, 5-diphenyltetrazolium bromide) assay as described by Mosman (65).
The equation used for calculation of percentage cytotoxicity:

$$(1 - (\mathrm{av}(\mathrm{x}) / (\mathrm{av}(\mathrm{NC}))) * 100 \qquad \text{Eq. (1)}$$

Where Av: average, X: absorbance of sample well measured at 595 nm with reference 690 nm, NC: absorbance of negative control measured at 595 nm with reference 690.

Determination of IC_{50} values

In case of highly active samples possessing ≥ 75 % cytotoxicity on different cancer cell lines, different concentrations were prepared for dose response studies. The results were used to calculate the IC_{50} values of each sample using probit analysis and utilizing the SPSS computer program (SPSS for windows, statistical analysis software package / version 9 / 1989 SPSS Inc., Chicago, USA).
